# Placental Chorangiosis: Increased Risk for Cesarean Section

**DOI:** 10.1155/2017/5610945

**Published:** 2017-05-21

**Authors:** Shariska S. Petersen, Raminder Khangura, Dmitry Davydov, Ziying Zhang, Roopina Sangha

**Affiliations:** ^1^Department of Women's Health Services, Henry Ford Hospital, Detroit, MI 48202, USA; ^2^Wayne State University School of Medicine, Detroit, MI 48202, USA; ^3^Department of Pathology, Cytopathology Laboratory, Henry Ford Hospital, Detroit, MI 48202, USA

## Abstract

We describe a patient with Class C diabetes who presented for nonstress testing at 36 weeks and 4 days of gestation with nonreassuring fetal heart tones (NRFHT) and oligohydramnios. Upon delivery, thrombosis of the umbilical cord was grossly noted. Pathological analysis of the placenta revealed chorangiosis, vascular congestion, and 40% occlusion of the umbilical vein. Chorangiosis is a vascular change of the placenta that involves the terminal chorionic villi. It has been proposed to result from longstanding, low-grade hypoxia in the placental tissue and has been associated with such conditions such as diabetes, intrauterine growth restriction (IUGR), and hypertensive conditions in pregnancy. To characterize chorangiosis and its associated obstetric outcomes we identified 61 cases of “chorangiosis” on placental pathology at Henry Ford Hospital from 2010 to 2015. Five of these cases were omitted due to lack of complete records. Among the 56 cases, the cesarean section rate was 51%, indicated in most cases for nonreassuring fetal status. Thus, we suggest that chorangiosis, a marker of chronic hypoxia, is associated with increased rates of cesarean sections for nonreassuring fetal status because of long standing hypoxia coupled with the stress of labor.

## 1. Introduction

Chorangiosis refers to the marked increase in the number of vascular channels in noninfarcted, nonischemic area of the placenta. The classic definition is more than 10 capillaries in more than 10 villi in several areas of placenta [1]. It is an uncommon finding that is widely described as a compensatory response to chronic hypoxia [1]; however, it is associated with common conditions including diabetes, hypertension, and tobacco use [2]. Using placental tissue oxygen index values, Suzuki et al. have shown an association between oxygen saturation of maternal blood in intervillous spaces and development of chorangiosis [2]. They postulate that low efficiency of oxygen transfer from maternal to fetal circulation facilitates vascular remodeling in adaptation to low oxygen supply, resulting in chorangiosis [2].

Umbilical vein thrombus is a rare occurrence and most occurrences are complications of cord compression and circulatory stasis [3]. Maternal diabetes as well as umbilical cord abnormalities including excessively long umbilical cords, true knots, and excessively twisted umbilical cords have also been associated with umbilical cord thrombus [3, 4]. Dussaux et al. report on a case of umbilical cord thrombus noted antenatally by power Doppler which resulted in intrauterine fetal death [5]. Thus, the finding of both umbilical cord thrombus and chorangiosis in a recent case prompted a review of our recent experience at Henry Ford Hospital.

## 2. Case Report

**Figure 1:**
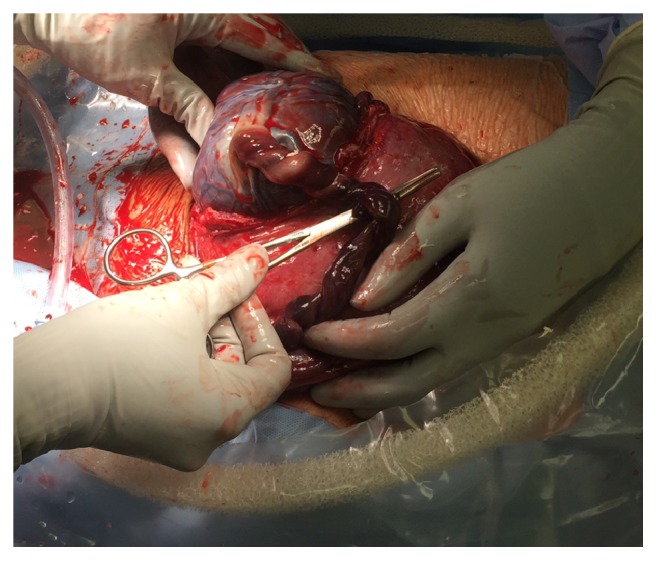
Gross umbilical cord at time of delivery.

A 38-year-old* gravida 7 para 5015 *patient with Class C diabetes and singleton pregnancy presented at 36 + 4/7 weeks of gestation for scheduled nonstress testing with the additional report of decreased fetal movement for the last day. The patient's blood glucose logs indicated sufficient glucose control and her hemoglobin A1c one month prior to presentation was 7.1%. Fetal growth was greater than the 98th percentile with an estimated fetal weight of 3616 grams and an amniotic fluid index of 22.1 cm nineteen days prior to presentation at 33 weeks and 6 days of gestation. Fetal umbilical Doppler studies were not indicated and patient was monitored with twice weekly nonstress tests, which were within normal limits. She was a nonsmoker and had an overweight BMI of 28.47 kg/m^2^. On presentation, bedside ultrasonography identified amniotic fluid level of 4.75 cm and the fetal heart rate tracing indicated irregular contractions with recurrent late-pattern decelerations from a baseline of* 150 *with moderate variability to 90 beats per minute lasting about one minute. Due to nonreassuring fetal status with oligohydramnios, delivery was affected by repeat low transverse cesarean section. At delivery, the umbilical cord was dusky and thrombus was palpable (*Figure 1*). The male infant weighed 4015 g. Resuscitation was rapid with Apgar scores 8 and 9 at 1 and 5 minutes, and other than transient hypoglycemia, the neonatal course was uneventful. The maternal postpartum course was also uncomplicated.

**Figure 2:**
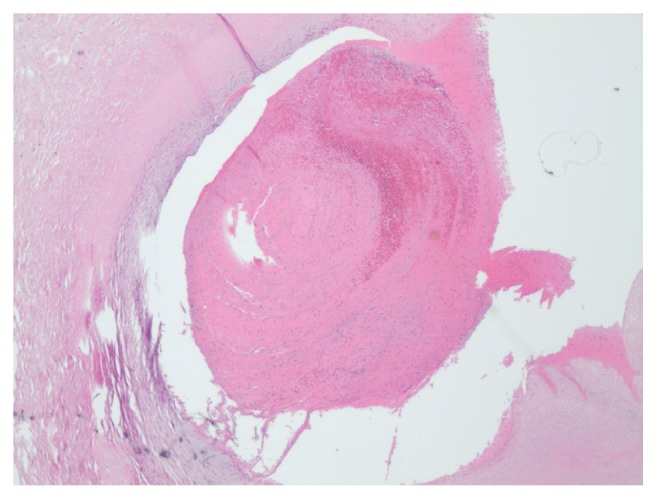
Cross section of umbilical vein with 40% thrombus occluding the vessel.

**Figure 3:**
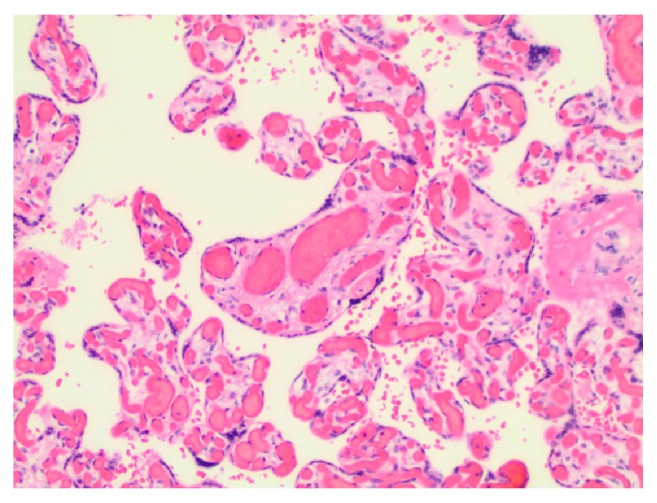
Chorionic villi with evidence of chorangiosis; more than 10 capillaries in at least 10 terminal villi in low-power fields (10x).

The placenta weighed 501 grams and the disc grossly appeared normal; however, the entire length of the cord appeared dark and mottled, showing signs of thrombosis. The pathological report described a three-vessel umbilical cord with subocclusive thrombosis of the umbilical vein, narrowing the vascular lumen by 40% (*Figure 2*). The fetal membranes were unremarkable. There were vascular congestion of the chorionic plate and a small 10 mm diameter area of subchorionic hemorrhage. Due to greater than the 10 capillaries per high power field of the chorionic villi, chorangiosis was identified (*Figure 3*).

## 3. Chart Review Methods

Approval for the chart review was obtained from the Institutional Review Board at Henry Ford Hospital. Patient medical record numbers were identified from the Pathology Department case index file from 2010 to 2015. Sixty-one cases of “chorangiosis” were identified by five staff cytopathologists: 5 cases were excluded due to incomplete medical record information. A descriptive analysis of the remaining 56 cases was completed using data from the integrated electronic health record within the Henry Ford Health System. We determined maternal age, BMI prior to twenty weeks of gestation, smoking status, maternal health conditions including presence of hypertensive conditions and diabetes, antenatal fetal issues including intrauterine growth restriction, gestational age at delivery, mode of delivery, indication for cesarean delivery (if performed), Apgar score, and neonatal weight through retrospective chart review.

## 4. Results

The demographic profile of the 56 cases with complete information is summarized in Table 1. The profile is reflective of the patients serviced by Henry Ford Hospital: obesity is a growing problem in southeast Michigan and tobacco use amongst low-income young women is a continuing national issue. As a referral hospital, we have a rich population with chronic diseases in pregnancy and patients in their late reproductive years. The obstetric outcomes are summarized in Table 2. The rate of cesarean section was noted to be 51.8% with the most common indication being nonreassuring fetal heart rate. Majority of neonates were delivered at term with the average gestational age of 37.5 weeks.

While the neonatal outcomes summarized in Table 3 are largely reassuring, the occurrence of 11% of patients with growth restriction, one with intrauterine fetal demise, and one neonatal death, support the idea that chorangiosis is a pathologic entity rather than an incidental observation. Chorangiosis was only associated with umbilical cord thrombus of one placenta in this study.

## 5. Discussion

The etiology and clinical associations of chorangiosis are not well understood; however, this finding is associated with fetal, maternal, and placental disorders including preeclampsia, diabetes, hypertension, major congenital anomalies, air pollution, and smoking [6] and has been correlated with fetal morbidity and mortality rates as high as 42% [1]. In our recent experience, the pregnancy outcomes are much improved over those suggested by Altshuler in 1984. The average placental/birth weight ratio for this cohort, 0.17, is considered normal in studies that correlate high placenta/birth weight ratio to adverse perinatal outcomes [7, 8]. Adverse events in our review, such as neonatal death at 22 weeks of gestation, are explainable by prematurity without need to invoke chronic hypoxia.

The rate of cesarean section in this cohort was 51.8%, which was much higher than the institution rate of 29% of 15,431 deliveries during this timeframe. The most common indication for cesarean section in this cohort was fetal heart rate abnormalities. Chorangiosis was only associated with cord thrombus in the sentinel case, suggesting that the two events are separate. This is supported by a study that found no correlation between fetal vessel thrombosis and chorangiosis in twin placentas [9]. Furthermore, chorangiosis has been associated with multiple umbilical cord complications but not a single complication [4] such as umbilical cord thrombus as seen on our sentinel case. We suspect that the umbilical cord thrombus was likely related to the patient's diabetes, since infants of diabetic mothers have increased *α* 2-antiplasmin and decreased fibrinolysin activity resulting in higher risk of thrombus formation [5]. We postulate that the umbilical vein thrombus was recently formed, coinciding with the decreased fetal movement the patient experienced and that the chorangiosis was resultant of a long standing hypoxia.

Over two-thirds of our cohort reported being former or current smokers suggesting that chorangiosis might be a compensatory mechanism for maternal hypoxia as suggested by Akbulut et al. In addition, the rate of maternal obesity, 45%, is also exaggerated in this group. This increased rate of obesity and smoking may suggest an association between obesity, smoking, and decreased efficiency of oxygen transfer to the fetal circulation resulting in chorangiosis. In our cohort, chorangiosis did not have the reported strong association with hypertensive disorders, diabetes, or preterm deliveries. However, we are limited by the inability to compare the incidence of these comorbidities to patients with normal placentas. At our institution, placentas are sent to pathology only if there is a pregnancy/delivery complication or a clinical observation such as the cord thrombosis in the sentinel case. Thus, we do not have “normal placenta” controls, nor can we estimate the prevalence of chorangiosis in our population. An editorial by Schwartz suggests that the existence of chorangiosis in many infants with hypoxia will remain unknown because the placenta has often been discarded [10].

We have shown that cesarean delivery is enriched in patients with placental chorangiosis; however, chorangiosis is not the direct cause. Chorangiosis is a placental marker of antepartum low-grade chronic hypoxia; thus, clinical correlation of entities that may contribute to hypoxia is suggested. Our review has identified smoking history and obesity to be associated with placental chorangiosis. Obesity has been long associated with increased cesarean section rates [11]. We postulate that obesity and smoking may contribute to low-grade hypoxia resulting in chorangiosis, and this chronic hypoxic state when coupled with the stress of labor can result in nonreassuring fetal status and increased rates of cesarean delivery.

## Figures and Tables

**Table 1:** Demographic profile and antenatal characteristics.

		Patients (*N* = 56)	Percentage of total (%)
Age	<18 years old	1	1.8
	18–34 years old	44	78.6
	≥35 years old	11	19.6

Race	Black	17	30.4
	White	30	53.6
	Latina	5	8.9
	Unknown	4	7.1

Body mass index (kg/m^2^)	<18.5	1	1.8
	18.5–24.9	17	30.4
	25.0–29.9	13	23.2
	>30	25	44.6
	Unknown	1	1.8

Tobacco use	Current	5	8.9
	Former	37	66.1
	Never	12	21.4
	Unknown	2	3.6

Gestation	Singleton	47	83.9
	Twins	9	16.1

Parity	Primiparous	24	42.9
	Multiparous	32	57.1

Comorbid conditions	Hypertension	9	16.1
	Diabetes	6	10.7
	Intrauterine growth restriction	6	10.7

**Table 2:** Obstetrical outcomes.

		All patients	Percentage of total
		*N* = 56	(%)
Gestational age at delivery	<37 completed weeks	10	17.8
	>37 weeks	46	82.1

Mode of delivery	Vaginal	27	48.2
	Cesarean delivery	29	51.8

Cesarean delivery indication	Fetal heart rate abnormality	10	17.9
	Malpresentation	6	10.7
	Labor abnormality	3	5.4
	Prior cesarean delivery	7	12.5
	Other^1^	2	3.6

^1^One patient had cesarean delivery for active HSV lesion and the other for history of a 4th-degree perineal laceration.

**Table 3:** Neonatal outcomes.

	All neonates	All singletons	Twin gestation
	*N* = 65	*N* = 47	*N* = 18
Mean Apgar score at 1 minute	7.3	7.0	8.0
Mean Apgar score at 5 minutes	8.6	8.5	8.9
Mean gestational age (completed weeks)	37.5	37.9	36.4
Mean birth weight (grams)	2996.4	3189.1	2493.2
Mean placental weight (grams)	504	525.7	447.8
Placental/birth weight ratio	0.17	0.16	0.18
Born alive	63	45	18
Neonatal death	1	1	0
Intrauterine fetal demise	1	1	0
